# Advances in Breast Cancer Management and Extracellular Vesicle Research, a Bibliometric Analysis

**DOI:** 10.3390/curroncol28060382

**Published:** 2021-11-08

**Authors:** Ramon Handerson Gomes Teles, Rafael Sussumu Yano, Nicolas Jones Villarinho, Ana Sayuri Yamagata, Ruy Gastaldoni Jaeger, Patrick Meybohm, Malgorzata Burek, Vanessa Morais Freitas

**Affiliations:** 1 Laboratory of Tumor Microenvironment, Department of Cell and Developmental Biology, Institute of Biomedical Sciences (ICB), University of São Paulo, São Paulo 05508-000, Brazil; rafael.sussumu.yano@usp.br (R.S.Y.); villarinhonicolas@icb.usp.br (N.J.V.); ana.yamagata@usp.br (A.S.Y.); rgjaeger@usp.br (R.G.J.); vfreitas@usp.br (V.M.F.); 2 Department of Anaesthesiology, Intensive Care, Emergency and Pain Medicine, University Hospital Würzburg, 97080 Würzburg, Germany; Meybohm_P@ukw.de (P.M.); Burek_M@ukw.de (M.B.)

**Keywords:** breast cancer, metastasis, exosomes, extracellular vesicles, bibliometrics

## Abstract

Extracellular vesicles transport variable content and have crucial functions in cell–cell communication. The role of extracellular vesicles in cancer is a current hot topic, and no bibliometric study has ever analyzed research production regarding their role in breast cancer and indicated the trends in the field. In this way, we aimed to investigate the trends in breast cancer management involved with extracellular vesicle research. Articles were retrieved from Scopus, including all the documents published concerning breast cancer and extracellular vesicles. We analyzed authors, journals, citations, affiliations, and keywords, besides other bibliometric analyses, using R Studio version 3.6.2. and VOSviewer version 1.6.0. A total of 1151 articles were retrieved, and as the main result, our analysis revealed trending topics on biomarkers of liquid biopsy, drug delivery, chemotherapy, autophagy, and microRNA. Additionally, research related to extracellular vesicles in breast cancer has been focused on diagnosis, treatment, and mechanisms of action of breast tumor-derived vesicles. Future studies are expected to explore the role of extracellular vesicles on autophagy and microRNA, besides investigating the application of extracellular vesicles from liquid biopsies for biomarkers and drug delivery, enabling the development and validation of therapeutic strategies for specific cancers.

## 1. Introduction

Cancer is a disease characterized by the rapid growth and division of abnormal cells [1] and is considered a public health concern for being one of the major causes of death and the second among non-communicable diseases worldwide (8.2 million deaths) [2]. Among all cancer types, breast cancer is the most common among women and is also associated with the highest mortality rate [3]. In 2020, it was estimated that breast cancer may be accountable for 11.7% of new cases and 6.9% of cancer deaths [4]. Moreover, the mortality of this type of cancer increases when it progresses and becomes metastatic, reducing the chances of survival to 10% [5].

Metastasis is the main cause of death related to tumor progression and constitutes a great challenge in cancer treatment. Currently, the main steps of the metastatic cascade are well described [6,7]. It starts from the detachment of single tumor cells or clusters, which migrate in the solid tissue until they reach the lymphatic vessels. Then, the cells enter the bloodstream or directly penetrate a distant tissue from the lymphatic system. After crossing the endothelial barrier, extravasation, the survival cells establish themselves in a pre-metastatic niche [8]. Breast cancer cells have tropism for visceral organs, such as the lungs, bones, liver, and brain [9]. However, the metastatic cascade is highly inefficient, with an estimated loss of 99.99% of tumor cell viability during the process [10].

However, some details of the metastatic cascade remain unclear, and uncovering them are the goals of many research groups worldwide [11,12], for example, those researching the signaling process between the pre-metastatic niche and the primary tumor, as well as the interaction between the microenvironment and the circulating tumor cells [13,14]. It is believed that extracellular vesicles (EVs) may be involved in this communication. The vesicles are continuously released by all kinds of cells and exert a role in intercellular communication [15]. To date, tumor-derived EVs have been the subject of many investigations.

EVs carry RNAs, DNA, proteins, lipids, and other different cargos [15]. Their classification is mainly based on size and biogenesis: (1) apoptotic bodies are the largest, ranging from 50 to 5000 nm, and are shed from dying cells; (2) microvesicles range from 100 to 1000 nm and originate from the budding of the cytoplasmic membrane; exosomes are the smallest, ranging from 30 to 100 nm, and are released from multivesicular bodies [16,17,18].

The literature shows extracellular vesicle contributions to tumor growth and cell dissemination from a primary breast tumor. It has been observed that breast tumor cells under chemotherapy alter the released extracellular vesicular content and generate phenotypic changes in resident lung macrophages, providing an inflammatory profile and sharply increasing the formation of metastases by chemotaxis to circulating tumor cells [19]. Additionally, a study analyzed if the impairment of extracellular vesicle release could exert influence on the formation of metastases. The authors used a model of breast cancer transplanted in an animal knockout to neutral sphingomyelinase 2 (nSMase 2) and observed a significant reduction in the extracellular vesicle release and a correlation with the decrease in metastases formation [20].

Moreover, it has been reported that breast cancer extracellular vesicles are able to transfer miR-122 to brain and lung tissues, inhibiting the expression of the enzyme pyruvate kinase, from the glycolytic pathway, consequently decreasing the supply of glucose to cells that composes these organs. Thus, facilitating the installation of metastatic foci in these tissues due to the high availability of local glucose, favoring tumor metabolism, is partly dependent on glycolytic metabolism [21]. Extracellular vesicles have also been observed to be able to carry miR-181c from breast tumor cells that form brain metastasis and influence the leakage of metastatic cells into the brain parenchyma crossing the blood–brain barrier [22].

Notably, there is an increasing number of articles being published each year on breast cancer and extracellular vesicles [23]. Therefore, keeping a constant and appreciable update of its state of the art may be challenging. A method to solve this issue is to perform a bibliometric analysis on the current literature. Bibliometric analysis is a well-accepted statistical tool that gives an overview of a research field and may help the scientific community by providing concise results from the increasing number of published papers [24,25].

Bibliometric analysis produces objective and subjective results from massive data (hundreds of thousands) [26,27]. This technique is useful for deciphering and mapping the scientific knowledge accumulated throughout years of publications, revealing evolutionary nuances in well-established fields by rigorous statistical analysis [28]. Therefore, bibliometric studies can predict, in a highly reliable way, the advances in a research field, identify knowledge gaps, and indicate novel ideas for impactful investigations [29].

In this way, since Scopus is the largest science database regularly updated, besides being commonly accepted by other authors in impactful studies [30,31,32], we performed a bibliometric analysis on this database of all the articles that have been published regarding extracellular vesicles and breast cancer. Our study aimed to present the current scenario of the research on this theme and predict the next hotspots concerning it.

## 2. Materials and Methods

### 2.1. Literature Search

A literature search was performed in the Scopus Database, as we consider it a reliable source of bibliometric indicators, such as title and abstracts, citations, keywords, and other data mostly used in biomedical and biological studies based on this technique [33,34]. Two independent authors (R.H.G.T and R.S.Y.) conducted a search using the following keywords and Boolean operators: (“Exosomes” OR “Extracellular Vesicles” OR “Microvesicles”) AND “Breast Cancer”. All the documents published were analyzed, without filtering by year, language, or source (article, book, etc.), aiming to include everything that has been published on the theme, different from the approaches adopted in other bibliometric studies [35,36]. All data were retrieved in the same period (March, 2021) to avoid bias due to updates of documents in the database.

### 2.2. Statistical Analysis

The raw data were retrieved in files compacted in Bibtex, RIS, and CVS formats and analyzed in the software VOSviewer (version 1.6.0; Leiden University, Leiden, the Netherlands), allowing the creation of a keyword network and its clustering. Statistical analyses were performed both on R Studio (version 3.6.2), using the Bibliometrix package [37], and on GraphPad Prism (version 8), enabling the development of charts and a historical direct citation network.

## 3. Results

### Bibliometric Findings

The search resulted in 1151 documents, in which the first article involving exosomes and breast cancer was published in 1998; however, the first use of the term “exosomes” was in 1970 [38]. These documents accounted for 785 experimental and clinical articles and 295 reviews; the other 71 included different document types, such as entire books or individual chapters, conference papers, and editorial notes. The documents were published in 451 different sources, cited 41,363 times in total, and written by 5599 authors from 57 countries (Table 1).

**Figure 1:**
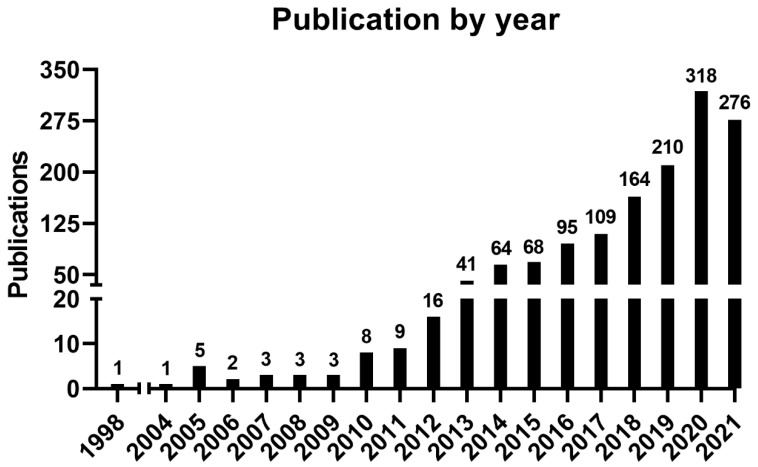
Number of publications by year.

**Figure 2:**
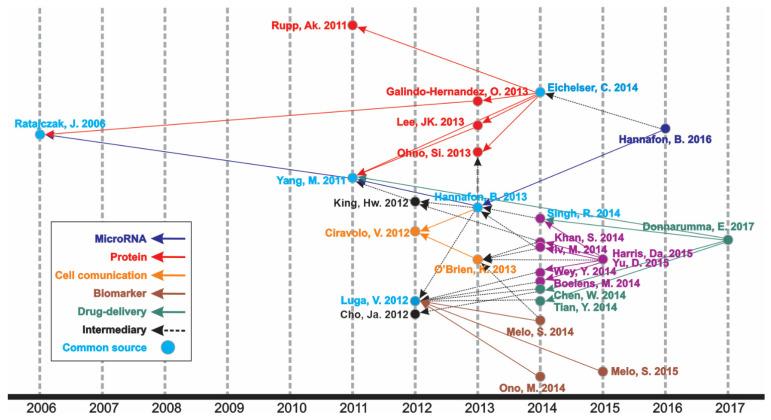
Historical direct citation network.

An increasing number of publications can be observed (Figure 1, Table S1, and Figure S1), wherein one article was published in 1998—the first one—and the curve reached its peak of 318 articles in 2020, with 276 more published articles until the moment of the data update (October 2021). The mean of the total citations per year normalized by the citable years is shown in Figure S1. It evidences some of the peaks of the citations that are correlated with the important articles published on the theme, which had a great impact and directed the research in the field, also making it possible to create a historiographic map to retrieve these impactful articles (Figure 2 and Table 2).

In the historiographic map, different groups of articles are observed indicating a scientific path throughout the years of publications on the theme, as indicated in Figure 2. Thus, the major path, meaning from the last impactful publication on the theme (in 2017) until the first one (in 2006), is constituted by five minor paths (MicroRNA, Protein, Cell communication, Biomarker, and Drug delivery), with some articles contributing to more than one minor path or presenting content of transition, respectively nominated as “Common source” and “Intermediary” paths. The studies identified by this analysis are listed in Table 2.

The most relevant source regarding the number of articles published (Table S2) was the *International Journal of Molecular Sciences* (38 documents, IF: 4.556), followed by *Oncotarget* (37 documents, IF: 5.168 in 2016) and *Scientific Reports* (32 documents, IF: 3.998). Taken together, these three journals represented 9.2% of all the publications on the theme.

However, the number of publications was not correlated with the citation number (Table S3). Thus, four journals (*Oncotarget, Plos One, Scientific Reports*, and *Cancer Research*) can be found in different positions in both tables, indicating that a higher citation number is not correlated with the higher impact of publications and citations of a journal. The top three most cited sources were “*Cancer Research*” (2116 citations, IF: 9.727), “*Nature*” (1491 citations, IF: 42.779), and “*Oncotarget*” (1447 citations, IF: 5.168 *). Coincidently, 8 out of 10 journals listed were also among the 50 journals that contributed the most, accounting for the top 100 most cited articles in extracellular vesicles and cancer [23]. “*Cancer Research*”, “*Plos One*”, “*Nature*”, and “*Cell and Nature Biology*” were the top 10 in both ranks. The top 10 journals comprise 27.5% of all citations.

The most cited article was “Glypican-1 identifies cancer exosomes and detects early pancreatic cancer”, written by Sonia A. Melo [50], published in “*Nature*”, and cited 1561 times. In this paper, a cell surface proteoglycan (glypican-1) was identified as being specifically enriched on cancer cell-derived exosomes, serving as a potential non-invasive diagnostic and screening tool to detect the early stages of pancreatic cancer (Table 3). Moreover, it is directly involved in the historical direct citation network (Figure 2), contributing to guiding other studies throughout the research on this theme. Yet, other important studies can be found through the career of the Top 10 most productive authors, which has Takahiro Ochiya leading the rank (Table S4).

**Figure 3:**
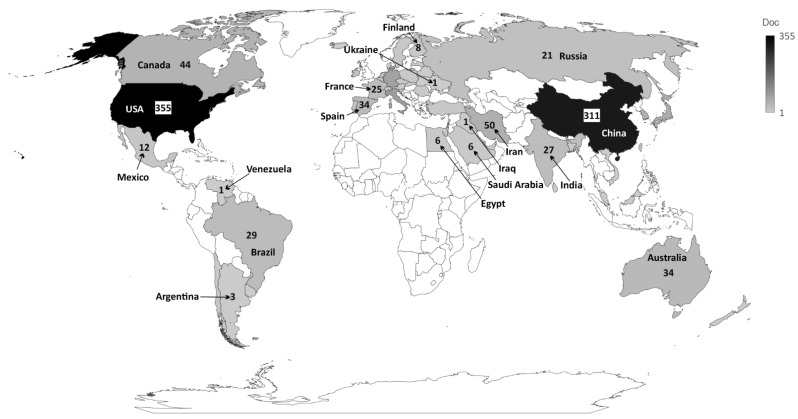
Countries that published most on the theme.

The institution presenting the highest production regarding the number of publications was “Cornell University”, USA, accounting for 36 publications, followed by “The University of California”, USA, with 35 publications and “Shahid Beheshti University of Medical Sciences”, Iran, with 33 publications. The top 10 most productive institutions are from the USA, Iran, and China, representing a closely distributed production (Table S5). Concerning the most productive country, the USA ranked as the first, in which all its institutions published 355 articles, followed by China, which published 311 articles, and Italy, which published 83 articles (Table S6 and Figure 3).

**Figure 4:**
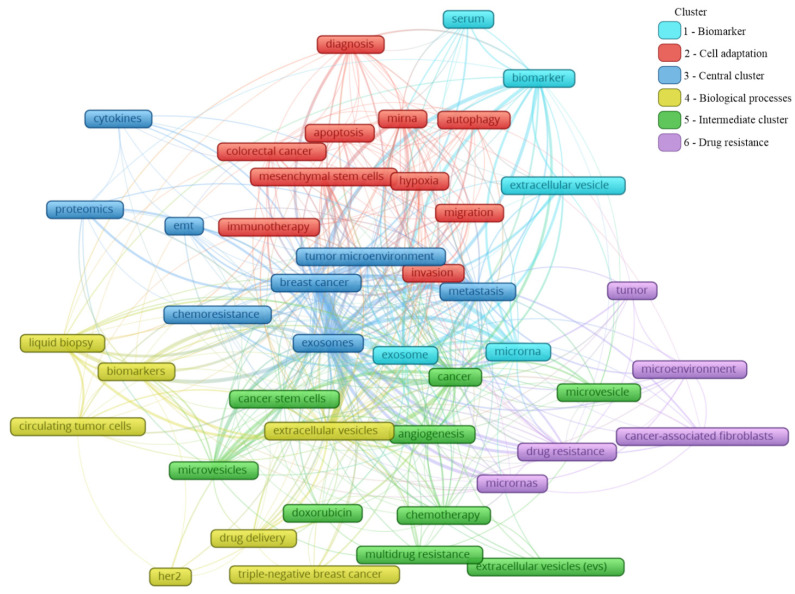
Main keyword clusters related to extracellular vesicles and breast cancer.

**Figure 5:**
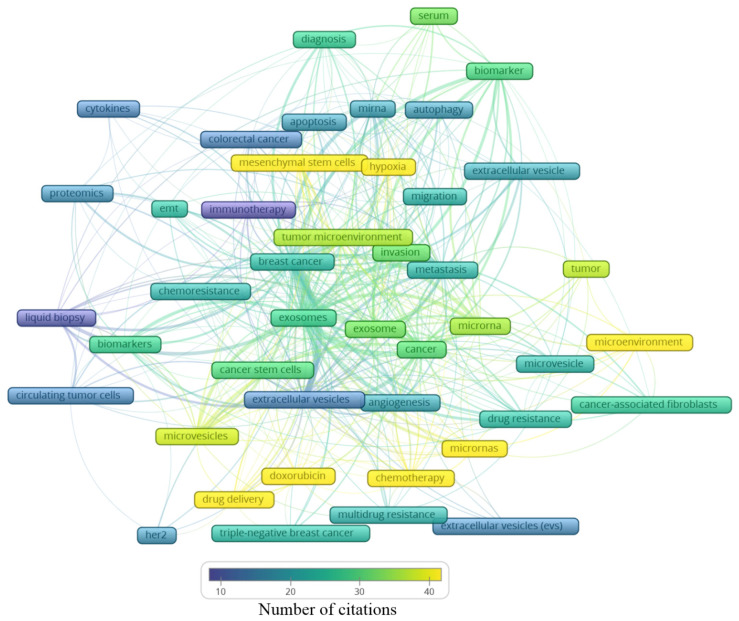
Average number of citations of the main keywords related to extracellular vesicles and breast cancer.

**Figure 6:**
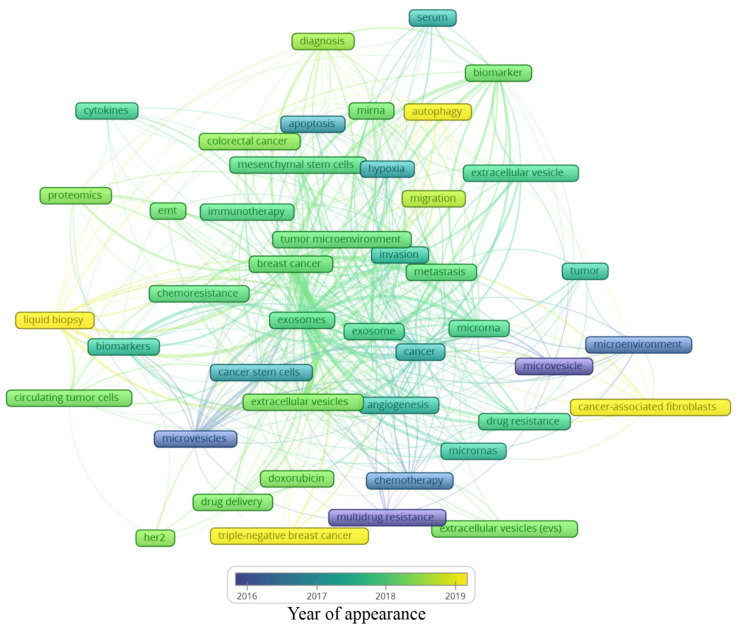
Average year of appearance of the main keywords related to extracellular vesicles and breast cancer.

A total of 2146 author keywords were retrieved, and 45 met the threshold of a minimum number of occurrences at 10 times cited (Table S7). These keywords were grouped into six clusters (Figure 4), presenting a different average of citation (Figure 5) and different years of appearance in the articles on the theme (Figure 6). The keywords with at least 10 occurrences, in the top 100 most cited articles in cancer and extracellular vesicles [23], roughly coincided with those we found. For instance, “microRNA”, “diagnostic biomarker”/“diagnosis” and “biomarker”, “proteomic analysis”/“proteomics”, “angiogenesis”, and “metastasis”. Interestingly, “chemotherapy” and “drug delivery”, included in our keyword network with approximately 40 citations, were not found in the mentioned previous study. This suggests that the research in breast cancer and extracellular vesicles may have been following an additional path for the last 4 years.

In Figure 4, the clusters are grouped by colors. From the cluster “1—Biomarker”, the keywords point to the microRNA of extracellular vesicles being used as biomarkers, mainly from the serum. The cluster “2—Cell adaptation” presents the process of autophagy and cell migration as being associated with a hypoxic microenvironment, probably having an influence on mesenchymal stem cells in colorectal cancer progression, and that could be treated with immunotherapy; additionally, miRNA is pointed as a strategy to be used in the diagnosis of colorectal cancer, as well as breast cancer. In cluster “6—Drug resistance”, an association between cancer-associated fibroblasts (CAFs) and microRNAs in the drug resistance process of the tumor microenvironment can be observed. The cluster “4—Biological processes” shows that drug delivery can be a strategy in the treatment of HER-2 and triple-negative breast cancer, and to improve the diagnosis accuracy, a liquid biopsy analyzing circulating tumor cells or using extracellular vesicles as biomarkers can constitute the next scientific direction on the theme. The cluster “5—Intermediate cluster” is an intermediate between the “6—Drug resistance” and the “4—Biological processes” clusters, in which the association between the tumor microenvironment and biological processes, such as angiogenesis, being mediated by microvesicles and not exclusively by exosomes, is displayed. Moreover, microvesicles and exosomes seem to influence other scenarios in cancer treatment, for example, the multidrug resistance to chemotherapeutics classically used in breast cancer, such as doxorubicin. In the final cluster, “3—Central cluster”, the main keywords are grouped, which are linked with all the clusters.

Based on these findings, it is reasonable to assume that exosomes might play a great role in the breast cancer microenvironment and metastasis development. Likewise, the chemoresistance process can be facilitated by these particles, representing a negative impact on the clinical setting. Proteomics was the methodology mostly used; it is associated with cytokine analysis, which is also linked with epithelial–mesenchymal transition, known to be essential to tumor progression and metastasis development.

Analyzing the clusters by average citation (Figure 5), the keywords most cited are related to drug delivery, in this case, doxorubicin, as well the influence of microRNAs on the tumor microenvironment, participating in the angiogenic process or even drug resistance. Moreover, hypoxia and mesenchymal stem cells showed an association with the tumor microenvironment, the invasion process, and metastasis. Exosomes and microvesicles were not often cited but are both in the center of the map as well as in each cluster, indicating their relevance in different scenarios regarding breast cancer.

Analyzing the year of appearance (Figure 6), the classic keywords on the theme, presenting themselves as the oldest, are “microvesicles”, “multidrug resistance”, and “microenvironment”. As the research was directed toward biological processes, other keywords were evidenced, such as “apoptosis”, “hypoxia”, and “angiogenesis”. Following the advances in the field, the next steps in treatment approaches were observed, for instance, “drug delivery”, “doxorubicin”, and “chemoresistance”. In recent years, a mixture of themes is present, with current hotspots “autophagy”, “liquid biopsy”, “cancer-associated fibroblasts”, and “triple-negative breast cancer”. The keywords and their different interactions in each map cannot be analyzed alone; it is important to consider all the links around each hotspot. In this manner, a general idea can be constructed around the newest keywords (Figure 6).

The keyword “autophagy” is linked to “extracellular vesicle”, “migration”, “tumor microenvironment”, and “miRNA”. Autophagy is a conserved catabolic process, in which cells degrade defective cytoplasmic molecules and organelles through lysosomal activity. This process is triggered by the communication of the cell with its microenvironment, being a response to external stress factors and an attempt to reach homeostasis [63]. Its vesicular machinery is involved in the exocytosis of extracellular vesicles through the fusion of the membrane of late endosomes with the cell membrane [64], establishing a route of communication between autophagic cancer cells and its milieu [63]. Autophagy can have a dual effect on cancer cells, suppressing (e.g., inhibit tumor growth) or promoting (e.g., maintenance of stem properties on cancer stem cells) tumorigenesis [65]. Hamurcu et al. (2017) observed that the triple-negative breast cancer cell line MDA-MB-231 has a higher rate of autophagy and expression of autophagy-related proteins LC3 and Beclin. Moreover, the downregulation of those proteins led to the suppression of autophagy, migration, proliferation, and colony formation potential and increased apoptosis [66]. Regarding the relation between “autophagy” and “miRNA”, the inhibitory potential of miRNA has been studied in this process. For example, the delivery of miRNA-376b through nanoparticles on breast cancer cells led to a reduction in tumor mass in a xenograft mice model and presented an even stronger effect when used in combination with cisplatin [67].

The keyword “cancer-associated fibroblasts” is linked to “metastasis”, “microvesicles”, “cancer”, and “microRNAs”. The tumor microenvironment is rich in stromal cells, such as fibroblasts, and those cells can be re-programmed due to the endocytosis of tumor-derived extracellular vesicles containing TGF-B1, TGF-B2, IL-6, MMP-2, and MMP-9 [68]. There is increasing evidence that these cancer-associated fibroblasts (CAFs) contribute to tumor progression and metastasis, placing it in the spotlight for novel anti-tumoral therapies [69]. CAF-derived EVs have different contents when compared with normal fibroblasts, for example, presenting the downregulation of miR-1-3p (a miRNA can inhibit the cell viability, invasion, migration, and epithelial–mesenchymal transition of breast cancer cells) when compared with normal fibroblasts. Then, Tao et al. (2020) transfected a miR-1-3p mimic into CAF-derived EVs, showing the potential of those cells in the delivery of the antineoplastic miRNA [70]. On the other hand, CAFs may modulate healthy and neoplastic cells through EVs, such as exosomes and microvesicles. For example, CD81+ exosomes are shed by CAFs and can be internalized by breast cancer cells, altering Wnt pathway molecules, which may contribute to an increase in cell protrusions and motility, favoring the metastasis cascade [71]. Moreover, exosomes seem to be involved in epithelial–mesenchymal transition, a key process in metastasis [72]. Sansone et al. (2017) showed the contribution of CAF-derived microvesicles in the acquisition of stemness properties of luminal breast cancer cells through the transference of miR-221, promoting resistance to hormonal therapies [73].

The keyword “triple-negative breast cancer” is linked to “extracellular vesicles”, “angiogenesis”, “drug delivery”, and “doxorubicin”. The classification of breast cancer can be based on the expression of the receptors for estrogen, progesterone, and HER2. The triple-negative (TN) subtype has a low expression of those hormone receptors and does not respond to hormone-based therapies, presenting a highly invasive nature, commonly evolving to metastatic disease [74]. Our group has demonstrated that the human TN cell line MDA-MB-231-derived EVs are more internalized by fibroblasts, and vice versa, than EVs derived from MCF-7, a luminal human cancer cell line [75]. TN breast cancer EVs can also interact with other cell types, such as endothelial cells, contributing to tumor progression [76]. In another study, it was observed that the association of breast cancer EVs and annexin 2A plays an important role in angiogenesis and the formation of a pre-metastatic niche through the activation of macrophages [77]. In a later publication of the same group, they correlated the previous association with the high incidence and aggressiveness of TN breast cancer in African American women [78]. EVs also show a promising antitumoral effect if employed as a drug delivery system. Gong et al. (2019) developed a delivery system using EVs secreted by human monocyte cells rich in a modified version of disintegrin and metalloproteinase-15 (A15), which binds to the integrin αvβ3 present in TN breast cancer cells. They tested the co-delivery of the chemotherapeutic agent doxorubicin and microRNA-159 and observed synergic anticancer effects both in in vitro and in vivo models of TN breast cancer [79]. In a similar study, Haney et al. (2019) demonstrated that monocyte EVs loaded with doxorubicin and paclitaxel induced the accumulation of those drugs inside of TN breast cancer cells in vitro and in vivo, increasing cytotoxicity and suppressing tumor formation, respectively [80].

Finally, the keyword “liquid biopsy” seems to be the most relevant among the newest keywords since it has many links, which are “breast cancer”, “biomarkers”, “diagnosis”, “extracellular vesicles”, “exosomes”, and “circulating tumor cells”. A liquid biopsy consists of a less invasive strategy for the detection of cancer relying on the molecular identification of the contents released from tumor cells, such as DNA, miRNA, and other biomarkers present in the blood, serum, and plasma [81]. Those molecules can be found free in those fluids or in circulating cancer cells or EVs. It has been demonstrated by several studies that EVs shed by breast cancer cells may contain stable miRNAs, such as miR-21 and miR-1246 in exosomes, constituting a potential tool in the future of breast cancer detection and diagnosis [39]. However, there is still no consensus in the scientific community about which specific miRNAs could be used for this purpose [82]. Hence, a liquid biopsy could allow the detection of cancer, prognosis, and could even guide therapy taking into account the molecular pattern of the tumor content present in the plasma of patients [82]. The growing research on non-coding RNA carried by extracellular vesicles goes beyond miRNAs, including ncRNAs, tRNA and tRNA fragments, Y RNA, piRNA, rRNA, and lncRNA [83], and requires a separate bibliometric study.

## 4. Strengths and Limitations

Our study provides a wide overview of the current literature regarding the influence of EVs on metastatic breast cancer, including not only information about authors, publishers, publication year, and number of citations but also the current hot topics and the trends predicted from them. However, since the data were collected only from Scopus, which contains a high degree of features that improved our analysis and constitutes the biggest repository of articles, other databases were not considered, which may represent a slight difference (but not significant) in the number of documents or other metrics.

## 5. Conclusions

Here, we quantitatively described the published literature related to extracellular vesicles and breast cancer and used these metrics to predict the future paths in the research on the oncology field. The journal with the highest number of publications on the subject was the “*International Journal of Molecular Sciences*” (*n* = 38), but the most cited was “*Cancer Research*” (*n* = 2116). The most productive author on this theme was “Melo, S.A.” (1561 citations), and the most cited article was by “Melo, et al.” (2015) [50] (*n* = 1561). The keyword networks revealed that researchers have been focusing not only on the mechanisms of action of breast tumor-derived vesicles but also on their implications in cancer treatment. Future studies are expected to explore the role of extracellular vesicles on autophagy and microRNA and the applications of extracellular vesicle knowledge from liquid biopsies for the development of better breast cancer biomarkers, contributing to personalized medicine. Additionally, the specificity of these vesicles could be explored as treatment, using them in biohybrid systems for precise drug delivery.

## Figures and Tables

**Table 1:** General information from the articles analyzed.

Documents	Components	Indexes
	Articles, reviews	1157
	Journals, books	451
	Author’s keywords	7924
	Period	1998–2021
	Average citations per document	35.75
**Authors**		
	Authors	5599
	Author appearances	8141
	Authors of single-authored documents	38
	Authors of multi-authored documents	5561
	Single-authored documents	43
	Documents per author	0.206
**Colaborations**		
	Authors per document	4.84
	Co-Authors per document	7.07
	Collaboration index	5.02

**Table 2:** Historical direct citation network studies.

Author	Article	Year	Total Citations	Ref.
**MicroRNA**				
Hannafon B.N., *Breast Cancer Res.*	Plasma exosome microRNAs are indicative of breast cancer	2016	257	[39]
Hannafon B.N., *Int. J. Mol. Sci.*	Intercellular communication by exosome-derived microRNAs in cancer	2013	375	[40]
Yang M., *Mol. Cancer*	Microvesicles secreted by macrophages shuttle invasion-potentiating microRNAs into breast cancer cells	2011	603	[41]
Ratajczac J., *Leukemia.*	Membrane-derived microvesicles: important and underappreciated mediators of cell-to-cell communication.	2006	1336	[42]
**Protein**				
Eichelser C., *Oncotarget*	Increased serum levels of circulating exosomal microRNA-373 in receptor-negative breast cancer patients	2014	251	[43]
Rupp A.K., *Gynecol. Oncol.*	Loss of EpCAM expression in breast cancer derived serum exosomes: role of proteolytic cleavage	2011	212	[44]
Galindo-Hernandez O., *Arch. Med. Res.*	Elevated concentration of microvesicles isolated from peripheral blood in breast cancer patients	2013	90	[45]
Lee J.K., *PLoS ONE*	Exosomes Derived from Mesenchymal Stem Cells Suppress Angiogenesis by Down-Regulating VEGF Expression in Breast Cancer Cells	2013	379	[46]
Ohno S.I., *Mol. Ther.*	Systemically injected exosomes targeted to EGFR deliver antitumor microRNA to breast cancer cells	2013	981	[47]
**Cell comunication**				
Hannafon B.N., *Int. J. Mol. Sci.*	Intercellular communication by exosome-derived microRNAs in cancer	2013	375	[40]
O’Brien K., *Eur. J. Cancer*	Exosomes from triple-negative breast cancer cells can transfer phenotypic traits representing their cells of origin to secondary cells	2013	173	[48]
Ciravolo V., *J. Cell Physiol.*	Potential role of HER2-overexpressing exosomes in countering trastuzumab-based therapy	2012	350	[49]
**Biomarker**				
Melo S.A., *Nature*	Glypican-1 identifies cancer exosomes and detects early pancreatic cancer	2015	1561	[50]
Ono M., *Sci. Signal*	Exosomes from bone marrow mesenchymal stem cells contain a microRNA that promotes dormancy in metastatic breast cancer cells	2014	433	[51]
Melo S.A., *Cancer Cell*	Cancer exosomes perform cell-independent microRNA biogenesis and promote tumorigenesis	2014	1074	[52]
Luga H.W., *Cell*	Exosomes mediate stromal mobilization of autocrine Wnt-PCP signaling in breast cancer cell migration	2012	939	[53]
**Drug delivery**				
Donnarumma E., *Oncotarget*	Cancer-associated fibroblasts release exosomal microRNAs that dictate an aggressive phenotype in breast cancer	2017	165	[54]
Singh R., *Mol. Cancer*	Exosome-mediated transfer of miR-10b promotes cell invasion in breast cancer.	2014	274	[55]
Chen W.X., *Tumor Biol.*	Exosomes from docetaxel-resistant breast cancer cells alter chemosensitivity by delivering microRNAs	2014	140	[56]
Tian Y., *Biomaterials*	A doxorubicin delivery platform using engineered natural membrane vesicle exosomes for targeted tumor therapy	2014	854	[57]
Yang M., *Mol. Cancer*	Microvesicles secreted by macrophages shuttle invasion-potentiating microRNAs into breast cancer cells	2011	603	[41]
**Intermediary**				
Eichelser C., *Oncotarget*	Increased serum levels of circulating exosomal microRNA-373 in receptor-negative breast cancer patients	2014	251	[43]
Hannafon B.N., *Int. J. Mol. Sci.*	Intercellular communication by exosome-derived microRNAs in cancer	2013	375	[40]
O’Brien K., *Eur. J. Cancer*	Exosomes from triple-negative breast cancer cells can transfer phenotypic traits representing their cells of origin to secondary cells	2013	173	[48]
King H.W., *BMC Cancer*	Hypoxic enhancement of exosome release by breast cancer cells	2012	650	[58]
Cho J.A., *Int. J. Oncol.*	Exosomes from breast cancer cells can convert adipose tissue-derived mesenchymal stem cells into myofibroblast-like cells	2012	339	[59]

**Table 3:** Top 10 most cited articles.

Document Title	Main Finding	Author	Source	Cited By
Glypican-1 identifies cancer exosomes and detects early pancreatic cancer [50]	Glypican-1-enriched exosomes as potential specific biomarkers for early detection of pancreatic cancer.	Melo, S.A. et al.	*Nature*, 2015, 523(7559), pp. 177–182	1561
Circulating microRNA in body fluid: A new potential biomarker for cancer diagnosis and prognosis [60]	Review of microRNA as a promissing non-invasive tool for prediction, prognosis, and diagnosis of early cancer.	Kosaka, N., Iguchi, H., Ochiya, T.	*Cancer Science*, 2010, 101(10), pp. 2087–2092	922
Membrane-derived microvesicles: Important and underappreciated mediators of cell-to-cell communication [42]	Insights into different aspects of microvesicle roles in different topics, such as carcinogenesis, coagulation, communication between cells, immune response, and modulation.	Ratajczak, J. et al.	*Leukemia*, 2006, 20(9), pp. 1487–1495	910
Cancer exosomes perform cell-independent microRNA biogenesis and promote tumorigenesis [52]	Cancer exosomes can modulate the cell transcriptome via miRNAs associated with RISC loading complex. They can also process pre-miRNAs into miRNAs independently of cells.	Melo, S.A. et al.	*Cancer Cell*, 2015, 26(5), pp. 707–721	801
Systemically injected exosomes targeted to EGFR deliver antitumor microRNA to breast cancer cells [47]	Engineering exosomes as a potential RNA drug delivery system, addressing exosomes with let-7a miRNA (tumor suppressor) to specifically target EGRP, which is generally high in tumor epithelial cells.	Ohno, S.I. et al.	*Molecular Therapy*, 2013, 21(1), pp. 185–191	779
Exosomes mediate stromal mobilization of autocrine Wnt-PCP signaling in breast cancer cell migration [53]	Exosomes derived from cancer-associated fibroblasts and L-cells have an autocrine influence on Wnt-PCP signaling, a factor that regulates and assists breast cancer cells in the motility and metastasis process.	Luga, V., et al.	*Cell*, 2012, 151(7), pp. 1542–1556	728
A doxorubicin delivery platform using engineered natural membrane vesicle exosomes for targeted tumor therapy [57]	Engineering of the exosome membrane from immature dendritic cells by fusion with the iRGD-targeting peptide for αV integrin, thereby creating a drug delivery system for the chemotherapeutic doxorubicin to tumor tissue.	Tian, Y., et al.	*Biomaterials*, 2014, 35(7), pp. 2383–2390	680
Integrating liquid biopsies into the management of cancer [61]	Insights into various tumor-derived materials that can be the target of liquid biopsies, with the focus on ctDNA, and the potential use of this screening to improve diagnostic performance and the treatment choice.	Siravegna, G., Marsoni, S., Siena, S., Bardelli, A.	*Nature Reviews Clinical Oncology*, 2017, 14(9), pp. 531–548	644
Hypoxic enhancement of exosome release by breast cancer cells [58]	The condition of hypoxia leads to an increase in an exosome that is enriched with miR-210 released by breast cancer cells. This factor could lead to promotion of tumour invasion, progression, angiogenesis, and endothelial activation.	King, H.W., Michael, M.Z., Gleadle, J.M.	*BMC Cancer*, 2012, 12, 421	486
Breast-cancer-secreted miR-122 reprograms glucose metabolism in premetastatic niche to promote metastasis [62]	A higher level of miR-122 secreted by breast cancer mediates a decrease in glucose uptake by healthy normal cells in the premetastatic niche, which favors the uptake of this nutrient by cancer cells during the metastasis process.	Fong, M.Y., et al.	*Nature Cell Biology*, 2015, 17(2), pp. 183–194	477

## Supplementary Materials

The following are available online at https://www.mdpi.com/article/10.3390/curroncol28060382/s1. Figure S1: Annual scientific production and average citation per year, Table S1: annual scientific production, Table S2: Journals with the highest number of publications, Table S3: Most cited sources, Table S4: Top 10 most productive authors publishing articles on the theme, Table S5: Top 10 most productive affiliations, Table S6: Top 10 countries that published on the theme, Table S7: List of keywords used in the maps of clusters.

## References

[1] Hanahan D., Weinberg R.A. (2011). Hallmarks of cancer: The next generation. Cell.

[2] Forouzanfar M.H., Afshin A., Alexander L.T., Biryukov S., Brauer M., Cercy K., Charlson F.J., Cohen A.J., Dandona L., Estep K. (2016). Global, regional, and national comparative risk assessment of 79 behavioural, environmental and occupational, and metabolic risks or clusters of risks, 1990–2015: A systematic analysis for the Global Burden of Disease Study 2015. Lancet.

[3] Harbeck N., Gnant M. (2017). Breast cancer. Lancet.

[4] Sung H., Ferlay J., Siegel R.L., Laversanne M., Soerjomataram I., Jemal A., Bray F. (2021). Global cancer statistics 2020: GLOBOCAN estimates of incidence and mortality worldwide for 36 cancers in 185 countries. CA Cancer J Clin..

[5] Guan X. (2015). Cancer metastases: Challenges and opportunities. Acta Pharm. Sin. B.

[6] Van Zijl F., Krupitza G., Mikulits W. (2011). Initial steps of metastasis: Cell invasion and endothelial transmigration. Mutat. Res./Rev. Mutat. Res..

[7] VanderVorst K., Dreyer C.A., Konopelski S.E., Lee H., Ho H.Y.H., Carraway K.L. (2019). Wnt/PCP signaling contribution to carcinoma collective cell migration and metastasis. Cancer Res..

[8] Demicheli R., Desmedt C., Piccart M., Biganzoli E. (2019). Tumor dormancy at bedside: A late awakening. Breast.

[9] Ursini-Siegel J., Siegel P.M. (2016). The influence of the pre-metastatic niche on breast cancer metastasis. Cancer Lett..

[10] Friberg S., Nystrom A. (2015). Cancer Metastases: Early Dissemination and Late Recurrences. Cancer Growth Metastasis.

[11] Fares J., Kanojia D., Rashidi A., Ulasov I., Lesniak M.S. (2020). Genes that Mediate Metastasis across the Blood–Brain Barrier. Trends Cancer.

[12] Follain G., Osmani N., Azevedo A.S., Allio G., Mercier L., Karreman M.A., Solecki G., Garcia Leòn M.J., Lefebvre O., Fekonja N. (2018). Hemodynamic Forces Tune the Arrest, Adhesion, and Extravasation of Circulating Tumor Cells. Dev. Cell.

[13] Peinado H., Zhang H., Matei I.R., Costa-silva B., Hoshino A., Rodrigues G., Psaila B., Kaplan R.N., Bromberg J.F., Kang Y. (2017). Pre-metastatic niches: Organ-specific homes for metastases. Nat. Publ. Gr..

[14] Qian C.N., Mei Y., Zhang J. (2017). Cancer metastasis: Issues and challenges. Chin. J. Cancer.

[15] Colombo M., Raposo G., Théry C. (2014). Biogenesis, secretion, and intercellular interactions of exosomes and other extracellular vesicles. Annu. Rev. Cell Dev. Biol..

[16] Hoshino A., Kim H.S., Bojmar L., Gyan K.E., Cioffi M., Hernandez J., Zambirinis C.P., Rodrigues G., Molina H., Heissel S. (2020). Extracellular Vesicle and Particle Biomarkers Define Multiple Human Cancers. Cell.

[17] Théry C., Witwer K.W., Aikawa E., Alcaraz M.J., Anderson J.D., Andriantsitohaina R., Antoniou A., Arab T., Archer F., Atkin-Smith G.K. (2018). Minimal information for studies of extracellular vesicles 2018 (MISEV2018): A position statement of the International Society for Extracellular Vesicles and update of the MISEV2014 guidelines. J. Extracell. Vesicles.

[18] Medina C.B., Mehrotra P., Arandjelovic S., Perry J.S.A., Guo Y., Morioka S., Barron B., Walk S.F., Ghesquière B., Krupnick A.S. (2020). Metabolites released from apoptotic cells act as tissue messengers. Nature.

[19] Keklikoglou I., Cianciaruso C., Güç E., Squadrito M.L., Spring L.M., Tazzyman S., Lambein L., Poissonnier A., Ferraro G.B., Baer C. (2019). Chemotherapy elicits pro-metastatic extracellular vesicles in breast cancer models. Nat. Cell Biol..

[20] Kosaka N., Iguchi H., Hagiwara K., Yoshioka Y., Takeshita F., Ochiya T. (2013). Neutral Sphingomyelinase 2 (nSMase2)-dependent Exosomal Transfer of Angiogenic MicroRNAs Regulate Cancer Cell Metastasis. J. Biol. Chem..

[21] Zhou W., Fong M.Y., Min Y., Somlo G., Liu L., Palomares M.R., Yu Y., Chow A., O’Connor S.T.F., Chin A.R. (2014). Cancer-Secreted miR-105 destroys vascular endothelial barriers to promote metastasis. Cancer Cell.

[22] Tominaga N., Kosaka N., Ono M., Katsuda T., Yoshioka Y., Tamura K., Lötvall J., Nakagama H., Ochiya T. (2015). Brain metastatic cancer cells release microRNA-181c-containing extracellular vesicles capable of destructing blood-brain barrier. Nat. Commun..

[23] Shi S., Gao Y., Liu M., Bu Y., Wu J., Tian J., Zhang J. (2020). Top 100 most-cited articles on exosomes in the field of cancer: A bibliometric analysis and evidence mapping. Clin. Exp. Med..

[24] Zyoud S.H., Al-Jabi S.W. (2020). Mapping the situation of research on coronavirus disease-19 (COVID-19): A preliminary bibliometric analysis during the early stage of the outbreak. BMC Infect. Dis..

[25] Manoel Alves J., Handerson Gomes Teles R., do Valle Gomes Gatto C., Muñoz V.R., Regina Cominetti M., Garcia de Oliveira Duarte A.C. (2019). Mapping Research in the Obesity, Adipose Tissue, and MicroRNA Field: A Bibliometric Analysis. Cells.

[26] Broadus R.N. (1987). Toward a definition of “bibliometrics”. Scientometrics.

[27] Ellegaard O., Wallin J.A. (2015). The bibliometric analysis of scholarly production: How great is the impact?. Scientometrics.

[28] Donthu N., Kumar S., Mukherjee D., Pandey N., Lim W.M. (2021). How to conduct a bibliometric analysis: An overview and guidelines. J. Bus. Res..

[29] Verma S., Gustafsson A. (2020). Investigating the emerging COVID-19 research trends in the field of business and management: A bibliometric analysis approach. J. Bus. Res..

[30] Teles R.H.G., Moralles H.F., Cominetti M.R. (2018). Global trends in nanomedicine research on triple negative breast cancer: A bibliometric analysis. Int. J. Nanomed..

[31] Gao Y., Shi S., Ma W., Chen J., Cai Y., Ge L., Li L., Wu J., Tian J. (2019). Bibliometric analysis of global research on PD-1 and PD-L1 in the field of cancer. Int. Immunopharmacol..

[32] Kamdem J.P., Duarte A.E., Ibrahim M., Lukong K.E., Barros L.M., Roeder T. (2020). Bibliometric analysis of personalized humanized mouse and Drosophila models for effective combinational therapy in cancer patients. Biochim. Biophys. Acta Mol. Basis Dis..

[33] Özen Çınar İ. (2020). Bibliometric analysis of breast cancer research in the period 2009–2018. Int. J. Nurs. Pract..

[34] Akmal M., Hasnain N., Rehan A., Iqbal U., Hashmi S., Fatima K., Farooq M.Z., Khosa F., Siddiqi J., Khan M.K. (2020). Glioblastome Multiforme: A Bibliometric Analysis. World Neurosurg..

[35] Stout N.L., Alfano C.M., Belter C.W., Nitkin R., Cernich A., Lohmann Siegel K., Chan L. (2018). A Bibliometric Analysis of the Landscape of Cancer Rehabilitation Research (1992–2016). J. Natl. Cancer Inst..

[36] Liu W., Wu L., Zhang Y., Shi L., Yang X. (2020). Bibliometric analysis of research trends and characteristics of oral potentially malignant disorders. Clin. Oral Investig..

[37] Aria M., Cuccurullo C. (2017). Bibliometrix: An R-tool for comprehensive science mapping analysis. J. Informetr..

[38] Fox A.S., Yoon S.B. (1970). DNA-induced transformation in Drosophila: Locus-specificity and the establishment of transformed stocks. Proc. Natl. Acad. Sci. USA.

[39] Hannafon B.N., Trigoso Y.D., Calloway C.L., Zhao Y.D., Lum D.H., Welm A.L., Zhao Z.J., Blick K.E., Dooley W.C., Ding W.Q. (2016). Plasma exosome microRNAs are indicative of breast cancer. Breast Cancer Res..

[40] Hannafon B.N., Ding W.Q. (2013). Intercellular communication by exosome-derived microRNAs in cancer. Int. J. Mol. Sci..

[41] Yang M., Chen J., Su F., Yu B., Su F., Lin L., Liu Y., Huang J.D., Song E. (2011). Microvesicles secreted by macrophages shuttle invasion-potentiating microRNAs into breast cancer cells. Mol. Cancer.

[42] Ratajczak J., Wysoczynski M., Hayek F., Janowska-Wieczorek A., Ratajczak M.Z. (2006). Membrane-derived microvesicles: Important and underappreciated mediators of cell-to-cell communication. Leukemia.

[43] Eichelser C., Stückrath I., Müller V., Milde-Langosch K., Wikman H., Pantel K., Schwarzenbach H. (2014). Increased serum levels of circulating exosomal microRNA-373 in receptor-negative breast cancer patients. Oncotarget.

[44] Rupp A.K., Rupp C., Keller S., Brase J.C., Ehehalt R., Fogel M., Moldenhauer G., Marmé F., Sültmann H., Altevogt P. (2011). Loss of EpCAM expression in breast cancer derived serum exosomes: Role of proteolytic cleavage. Gynecol. Oncol..

[45] Galindo-Hernandez O., Villegas-Comonfort S., Candanedo F., González-Vázquez M.C., Chavez-Ocaña S., Jimenez-Villanueva X., Sierra-Martinez M., Salazar E.P. (2013). Elevated concentration of microvesicles isolated from peripheral blood in breast cancer patients. Arch. Med. Res..

[46] Lee J.K., Park S.R., Jung B.K., Jeon Y.K., Lee Y.S., Kim M.K., Kim Y.G., Jang J.Y., Kim C.W. (2013). Exosomes derived from mesenchymal stem cells suppress angiogenesis by down-regulating VEGF expression in breast cancer cells. PLoS ONE.

[47] Ohno S.I., Takanashi M., Sudo K., Ueda S., Ishikawa A., Matsuyama N., Fujita K., Mizutani T., Ohgi T., Ochiya T. (2013). Systemically injected exosomes targeted to EGFR deliver antitumor microrna to breast cancer cells. Mol. Ther..

[48] O’Brien K., Rani S., Corcoran C., Wallace R., Hughes L., Friel A.M., McDonnell S., Crown J., Radomski M.W., O’Driscoll L. (2013). Exosomes from triple-negative breast cancer cells can transfer phenotypic traits representing their cells of origin to secondary cells. Eur. J. Cancer.

[49] Ciravolo V., Huber V., Ghedini G.C., Venturelli E., Bianchi F., Campiglio M., Morelli D., Villa A., Della Mina P., Menard S. (2012). Potential role of HER2-overexpressing exosomes in countering trastuzumab-based therapy. J. Cell. Physiol..

[50] Melo S.A., Luecke L.B., Kahlert C., Fernandez A.F., Gammon S.T., Kaye J., LeBleu V.S., Mittendorf E.A., Weitz J., Rahbari N. (2015). Glypican-1 identifies cancer exosomes and detects early pancreatic cancer. Nature.

[51] Ono M., Kosaka N., Tominaga N., Yoshioka Y., Takeshita F., Takahashi R.U., Yoshida M., Tsuda H., Tamura K., Ochiya T. (2014). Exosomes from bone marrow mesenchymal stem cells contain a microRNA that promotes dormancy in metastatic breast cancer cells. Sci. Signal..

[52] Melo S.A., Sugimoto H., Connell J.T.O., Kato N., Vidal A., Qiu L., Vitkin E., Perelman L.T., Melo C.A., Lucci A. (2015). Cancer exosomes perform Cell-independent MicroRNA biogenesis and promote tumorigenesis. Cancer Cell.

[53] Luga V., Zhang L., Viloria-Petit A.M., Ogunjimi A.A., Inanlou M.R., Chiu E., Buchanan M., Hosein A.N., Basik M., Wrana J.L. (2012). Exosomes mediate stromal mobilization of autocrine Wnt-PCP signaling in breast cancer cell migration. Cell.

[54] Donnarumma E., Fiore D., Nappa M., Roscigno G., Adamo A., Iaboni M., Russo V., Affinito A., Puoti I., Quintavalle C. (2017). Cancer-associated fibroblasts release exosomal microRNAs that dictate an aggressive phenotype in breast cancer. Oncotarget.

[55] Singh R., Pochampally R., Watabe K., Lu Z., Mo Y.Y. (2014). Exosome-mediated transfer of miR-10b promotes cell invasion in breast cancer. Mol. Cancer.

[56] Chen W.X., Cai Y.Q., Lv M.M., Chen L., Zhong S.L., Ma T.F., Zhao J.H., Tang J.H. (2014). Exosomes from docetaxel-resistant breast cancer cells alter chemosensitivity by delivering microRNAs. Tumor Biol..

[57] Tian Y., Li S., Song J., Ji T., Zhu M., Anderson G.J., Wei J., Nie G. (2014). A doxorubicin delivery platform using engineered natural membrane vesicle exosomes for targeted tumor therapy. Biomaterials.

[58] King H.W., Michael M.Z., Gleadle J.M. (2012). Hypoxic enhancement of exosome release by breast cancer cells. BMC Cancer.

[59] Cho J.A., Park H., Lim E.H., Lee K.W. (2012). Exosomes from breast cancer cells can convert adipose tissue-derived mesenchymal stem cells into myofibroblast-like cells. Int. J. Oncol..

[60] Kosaka N., Iguchi H., Ochiya T. (2010). Circulating microRNA in body fluid: A new potential biomarker for cancer diagnosis and prognosis. Cancer Sci..

[61] Siravegna G., Marsoni S., Siena S., Bardelli A. (2017). Integrating liquid biopsies into the management of cancer. Nat. Rev. Clin. Oncol..

[62] Fong M.Y., Zhou W., Liu L., Alontaga A.Y., Chandra M., Ashby J., Chow A., O’Connor S.T.F., Li S., Chin A.R. (2015). Breast-cancer-secreted miR-122 reprograms glucose metabolism in premetastatic niche to promote metastasis. Nat. Cell Biol..

[63] Xu J., Camfield R., Gorski S.M. (2018). The interplay between exosomes and autophagy—partners in crime. J. Cell Sci..

[64] Buratta S., Tancini B., Sagini K., Delo F., Chiaradia E., Urbanelli L., Emiliani C. (2020). Lysosomal exocytosis, exosome release and secretory autophagy: The autophagic- and endo-lysosomal systems go extracellular. Int. J. Mol. Sci..

[65] Yun C.W., Lee S.H. (2018). The roles of autophagy in cancer. Int. J. Mol. Sci..

[66] Hamurcu Z., Delibaşı N., Geçene S., Şener E.F., Dönmez-Altuntaş H., Özkul Y., Canatan H., Ozpolat B. (2018). Targeting LC3 and Beclin-1 autophagy genes suppresses proliferation, survival, migration and invasion by inhibition of Cyclin-D1 and uPAR/Integrin β1/Src signaling in triple negative breast cancer cells. J. Cancer Res. Clin. Oncol..

[67] Unal O., Akkoc Y., Kocak M., Nalbat E., Dogan-Ekici A.I., Yagci Acar H., Gozuacik D. (2020). Treatment of breast cancer with autophagy inhibitory microRNAs carried by AGO2-conjugated nanoparticles. J. Nanobiotechnology.

[68] Heneberg P. (2016). Paracrine tumor signaling induces transdifferentiation of surrounding fibroblasts. Crit. Rev. Oncol. Hematol..

[69] Sahai E., Astsaturov I., Cukierman E., DeNardo D.G., Egeblad M., Evans R.M., Fearon D., Greten F.R., Hingorani S.R., Hunter T. (2020). A framework for advancing our understanding of cancer-associated fibroblasts. Nat. Rev. Cancer.

[70] Tao S., Li H., Ma X., Ma Y., He J., Gao Y., Li J. (2020). Elevating microRNA-1-3p shuttled by cancer-associated fibroblasts-derived extracellular vesicles suppresses breast cancer progression and metastasis by inhibiting GLIS1. Cancer Gene Ther..

[71] Luga V., Wrana J.L. (2013). Tumor-stroma interaction: Revealing fibroblast-secreted exosomes as potent regulators of Wnt-planar cell polarity signaling in cancer metastasis. Cancer Res..

[72] Greening D.W., Gopal S.K., Mathias R.A., Liu L., Sheng J., Zhu H.J., Simpson R.J. (2015). Emerging roles of exosomes during epithelial-mesenchymal transition and cancer progression. Semin. Cell Dev. Biol..

[73] Sansone P., Berishaj M., Rajasekhar V.K., Ceccarelli C., Chang Q., Strillacci A., Savini C., Shapiro L., Bowman R.L., Mastroleo C. (2017). Evolution of Cancer Stem-like Cells in Endocrine-Resistant Metastatic Breast Cancers is Mediated by Stromal Microvesicles. Cancer Res..

[74] Medina M.A., Oza G., Sharma A., Arriaga L.G., Hernández J.M.H., Rotello V.M., Ramirez J.T. (2020). Triple-negative breast cancer: A review of conventional and advanced therapeutic strategies. Int. J. Environ. Res. Public Health.

[75] Silva T.A., Smuczek B., Valadão I.C., Dzik L.M., Iglesia R.P., Cruz M.C., Zelanis A., de Siqueira A.S., Serrano S.M.T., Goldberg G.S. (2016). AHNAK enables mammary carcinoma cells to produce extracellular vesicles that increase neighboring fibroblast cell motility. Oncotarget.

[76] Kikuchi S., Yoshioka Y., Prieto-Vila M., Ochiya T. (2019). Involvement of extracellular vesicles in vascular-related functions in cancer progression and metastasis. Int. J. Mol. Sci..

[77] Yang G., Sau C., Lai W., Cichon J., Li W. (2015). Exosomal annexin A2 promotes angiogenesis in breast cancer metastasis. Mol. Cancer Res..

[78] Chaudhary P., Gibbs L.D., Maji S., Lewis C.M., Suzuki S., Vishwanatha J.K. (2020). Serum exosomal-annexin a2 is associated with african-American triple-negative breast cancer and promotes angiogenesis. Breast Cancer Res..

[79] Gong C., Tian J., Wang Z., Gao Y., Wu X., Ding X., Qiang L., Li G., Han Z., Yuan Y. (2019). Functional exosome-mediated co-delivery of doxorubicin and hydrophobically modified microRNA 159 for triple-negative breast cancer therapy. J. Nanobiotechnology.

[80] Haney M.J., Zhao Y., Jin Y.S., Li S.M., Bago J.R., Klyachko N.L., Kabanov A.V., Batrakova E.V. (2020). Macrophage-Derived Extracellular Vesicles as Drug Delivery Systems for Triple Negative Breast Cancer (TNBC) Therapy. J. Neuroimmune Pharmacol..

[81] Poulet G., Massias J., Taly V. (2019). Liquid Biopsy: General Concepts. Acta Cytol..

[82] Ozawa P.M.M., Jucoski T.S., Vieira E., Carvalho T.M., Malheiros D., Ribeiro E.M.D.S.F. (2020). Liquid biopsy for breast cancer using extracellular vesicles and cell-free microRNAs as biomarkers. Transl. Res..

[83] Abramowicz A., Story M.D. (2020). The Long and Short of It: The Emerging Roles of Non-Coding RNA in Small Extracellular Vesicles. Cancers.

