# Supplementary material for: Advances in Breast Cancer Management and Extracellular Vesicle Research, a Bibliometric Analysis
Source: Curr Oncol. 2021 Nov 8;28(6):4504–20. doi: 10.3390/curroncol28060382 (PMC8628791; doi:10.3390/curroncol28060382)
Supplement: Supplementary file 1 [file curroncol-28-00382-s001.zip › curroncol-1438099-supplementary.pdf]

Current Oncology

Advances in Breast Cancer Management and Extracellular Vesicle Research, a Bibliometric Analysis

Ramon Handerson Gomes Teles, Rafael Sussumu Yano, Nicolas Jones Villarinho, Ana Sayuri Yamagata, Ruy Gastaldoni Jaeger, Patrick Meybohm, Malgorzata Burek and Vanessa Morais Freitas

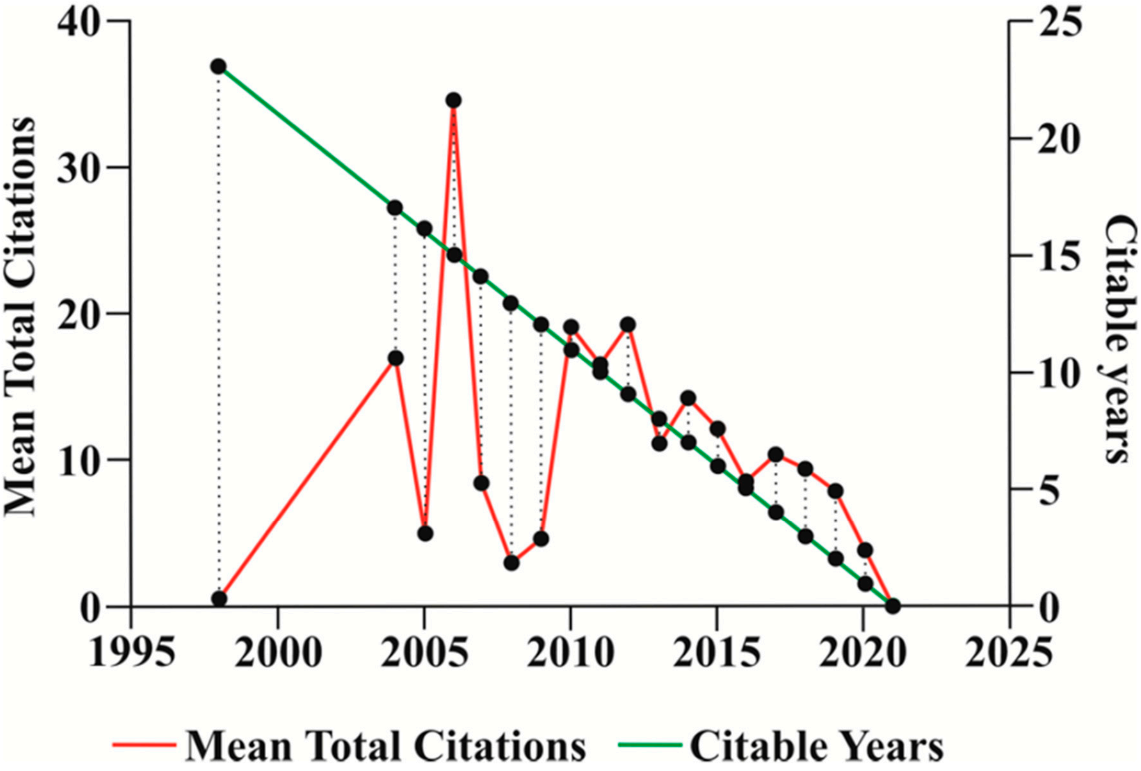

Figure S1. Annual Scientific Production and Average Citation per year .

Table S1. Annual Scientific Production.

Year	N	Mean Total Citation per Article	Mean Total Citation per Year	Citable Years
1998	1	16	0.69	23
2004	1	290	17.05	17
2005	5	80	5	16
2006	2	520.5	34.7	15
2007	3	123	8.78	14
2008	3	33.3	2.56	13
2009	3	55.6	4.63	12
2010	8	210.6	19.14	11
2011	9	165	16.5	10
2012	16	174.2	19.36	9
2013	41	89	11.12	8
2014	64	100.7	14.39	7
2015	68	73.5	12.26	6
2016	95	43.1	8.62	5

2017	109	41.9	10.48	4
2018	163	28.2	9.41	3
2019	205	15.8	7.94	2
2020	299	3.93	3.93	1
2021	56	0.05	-	0

Table S2. Journals with the highest number of publications.

ISSN	Journal	Documents	Impact Factor (2020)
1422-0067	International Journal of Molecular Sciences	38	4.556
1949-2553	Oncotarget	37	5.168*
2045-2322	Scientific Reports	32	3.998
2072-6694	Cancers	29	6.126
2234-943X	Frontiers in Oncology	23	4.848
1932-6203	Plos One	21	2.776
1538-7447	Cancer Research	18	9.727
1097-4652	Journal of Cellular Physiology	15	5.546
2001-3078	Journal of Extracellular Vesicles	15	14.976

*Last impact factor calculated in 2016.

Table S3. Most cited sources.

ISSN	Journal	Citations	Impact Factor (2019)
1538-7447	Cancer Research	2116	9.727
0028-0836	Nature	1491	42.779
1949-2553	Oncotarget	1447	5.168*
0092-8674	Cell	1310	38.637
0021-9258	Journal of Biological Chemistry	1304	4.238
1932-6203	Plos One	1300	2.740
1465-7392	Nature Cell Biology	1055	20.042
0950-9232	Oncogene	952	7.971
1078-0432	Clinical Cancer Research	817	10.107
2045-2322	Scientific Reports	798	3.998

*Last impact factor calculated in 2016.

Table S4. Top 10 most productive authors publishing articles on the theme.

Author	Affiliation	Documents	Citation
Ochiya, Takahiro	Tokyo Medical University, Tokyo, Japan	General	
		448	22790
h-index (2020)		On theme	
72		25	3229
Article most cited in general	Minimal information for studies of extracellular vesicles 2018 (MISEV2018): a position statement of the international society for extracellular vesicles and update of the MISEV2014 guidelines (17)	Journal of Extracellular Vesicles, 2018, 7(1), 1535750	1459
Article most cited on theme	Circulating microRNA in body fluid: A new potential biomarker for cancer diagnosis and prognosis (61)	Cancer Science, 2010, 101(10), pp.2087-2092	927
**Author**	**Affiliation**	**Documents**	**Citation**
Yoshioka, Yusuke	Tokyo Medical University, Tokyo, Japan	General	

		64	5258
h-index (2020)		On theme	
33		11	1100
Article most cited in general	Secretory mechanisms and intercellular transfer of microRNAs in living cells (63)	Journal of biological chemistry, 2010, 285(23), pp. 17442-17452	1233
Article most cited on theme	Exosomes from bone marrow mesenchymal stem cells contains a microRNA that promotes dormancy in metastatic breast cancer cells (48)	Science Signaling, 2014, 7(332), ra63	336
**Author**	Affiliation	Documents	Citation
Antonyak, Marc A.	Cornell University, Ithaca, United States	General	
		77	2793
h-index (2020)		On theme	
32		10	393
Article most cited in general	Cancer cell-derived microvesicles induce transformation by transferring tissue transglutaminase and fibronectin to recipient cells (64)	Proceedings of the national academy of sciences of the United States of America, 2011, 108(12), pp. 4852-4857	296
Article most cited on theme	RhoA triggers a specific signaling pathway that generates transforming microvesicles in cancer cells (65)	Oncogene, 2012, 31(45), pp. 4740-4749	158
**Author**	Affiliation	Documents	Citation
Cerione, Richard A.	Cornell University, Ithaca, United States	General	
		318	20420
h-index (2020)		On theme	
77		10	393
Article most cited in general	Sirt5 is a NAD-dependent protein lysine dematonylase and desuccinylase (66)	Science, 2011, 334(6057), pp. 806-809	740
Article most cited on theme	RhoA triggers a specific signaling pathway that generates transforming microvesicles in cancer cells (65)	Oncogene, 2012, 31(45), pp. 4740-4749	158
**Author**	**Affiliation**	**Documents**	**Citation**
Kosaka, Nobuyoshi	Tokyo Medical University, Tokyo, Japan	General	
		77	8454
h-index (2020)		On theme	
40		10	1932
Article most cited in general	Secretory mechanisms and intercellular transfer of microRNAs in living cells (63)	Journal of biological chemistry, 2010, 285(23), pp. 17442-17452	1233
Article most cited on theme	Circulating microRNA in body fluid: A new potential biomarker for cancer diagnosis and prognosis (61)	Cancer Science, 2010, 101(10), pp. 2087-2092	927
**Author**	**Affiliation**	**Documents**	**Citation**
O'Driscoll, Lorraine	Trinity College Dublin, Dublin, Ireland	General	
		121	9803
h-index (2020)		On theme	
43		10	748
Article most cited in general	Biological properties of extracellular vesicles and their physiological functions (67)	Journal of extracellular vesicles, 2015, 4(2015), pp. 1-60	1657

Article most cited on theme	Intracellular and extracellular microRNAs in breast cancer (68)	Clinical Chemistry, 2011, 57(1), pp. 18-32	175
**Author**	**Affiliation**	**Documents**	**Citation**
Tang, Jinhai	Jiangsu Province Hospital, Nanjing, China	General	
		226	5704
h-index (2020)		On theme	
41		10	595
Article most cited in general	MicroRNA-34a: A potential therapeutic target in human cancer (69)	Cell Death and Disease, 2014, 5(7), e1327	180
Article most cited on theme	Exosomes from drug-resistant breast cancer cells transmit chemoresistance by a horizontal transfer of microRNAs (49)	PLoS ONE, 2014, 9(4), e95240	127
**Author**	**Affiliation**	**Documents**	**Citation**
Tamkovich, Svetlana N	Altair Engineering India Pvt. Ltd., Bengaluru, India	General	
		59	955
h-index (2020)		On theme	
15		9	35
Article most cited in general	Circulating DNA and DNase activity in human blood (70)	Annals of the New York Academy of Sciences, 2006, 1075, pp. 191-196	126
Article most cited on theme	Exosomes: Generation, structure, transport, biological activity, and diagnostic application (71)	Biochemistry (Moscow) Supplement Series A: Membrane and Cell Biology, 2016, 10(3), pp. 163-173	15
**Author**	**Affiliation**	**Documents**	**Citation**
Kasimir-Bauer, Sabine	Universitäts Klinikum Essen und Medizinische Fakultät, Essen, Germany	General	
		128	3646
h-index (2020)		On theme	
32		8	103
Article most cited in general	Stem cell and epithelial-mesenchymal transition markers are frequently overexpressed in circulating tumor cells of metastatic breast cancer patients (72)	Breast Cancer Research, 2009, 11(4), R46	541
Article most cited on theme	The prognostic impact of soluble and vesicular HLA-G and its relationship to circulating tumor cells in neoadjuvant treated breast cancer patients (73)	Human Immunology, 2016, 77(9), pp. 791-799	44
**Author**	**Affiliation**	**Documents**	**Citation**
Laktionov, Pavel P.	Institute of Chemical Biology and Fundamental Medicine, SB RAS, Novosibirsk, Russia	General	
		193	2452
h-index (2020)		On theme	
25		8	36
Article most cited in general	Isolation of Extracellular Vesicles: General Methodologies and Latest Trends (74)	BioMed Research International, 2018, 2018, 8545347	238
Article most cited on theme	Exosomes: Generation, structure, transport, biological activity, and diagnostic application (71)	Biochemistry (Moscow) Supplement Series A: Membrane and Cell	15

		Biology, 2016, 10(3), pp. 163-173

Table S5. Top 10 most productive affiliations.

Institution	Origin	Documents
Cornell University	USA	36
University of California	USA	35
Shahid Beheshti University of Medical Sciences	Iran	33
Nanjing Medical University affiliated cancer hospital	China	28
Harvard Medical School	USA	27
National Cancer Center Research Institute	USA	26
Mashhad University of Medical Sciences	Iran	25
Nanjing Medical University	China	23
Fudan University	China	22
Tarbiat Modares University	Iran	20

Table S6. Top 10 countries that published on the theme.

Country	Doc	Country	Doc
USA	355	Iran	50
China	311	United Kingdom	50
Italy	83	South Korea	48
Germany	72	Canada	44
Japan	55	Australia	34

Table S7. List of keywords used in the maps of clusters.

ITEM	CLUSTER	LINKS	OCCURRENCES	AVG. CITATIONS	AVG. PUB. YEAR
APOPTOSIS	1	17	12	18	2016.92
AUTOPHAGY	1	18	10	17.80	2018.90
COLORECTAL CANCER	1	19	10	14.50	2018.30
DIAGNOSIS	1	20	22	26.32	2018.41
HYPOXIA	1	23	11	65.55	2017.09
IMMUNOTHERAPY	1	22	40	8.31	2017.88
INVASION	1	23	16	30.94	2017.44
MESENCHYMAL STEM CELLS	1	19	21	44.90	2017.90
MIGRATION	1	19	11	22.27	2018.55
MIRNA	1	32	36	18.58	2018.11
ANGIOGENESIS	2	25	29	19.55	2017.34
CANCER	2	35	120	29.68	2017.21
CANCER STEM CELLS	2	19	13	29.00	2017.00
CHEMOTHERAPY	2	22	19	43.37	2016.47
DOXORUBICIN	2	23	13	75.62	2018.23
EXTRACELLULAR VESICLES (EVS)	2	6	10	15.70	2018.20
MICROVESICLE	2	17	11	22.55	2014.91
MICROVESICLES	2	24	51	36.90	2016.27
MULTIDRUG RESISTANCE	2	14	12	22.08	2015.42
TRIPLE NEGATIVE BREAST CANCER	2	12	11	30.09	2018.91

BREAST CANCER	3	41	332	23.36	2018.03
CHEMORESISTANCE	3	17	18	22.11	2017.94
CYTOKINES	3	9	10	14.80	2017.70
EMT	3	11	12	23.75	2018.08
EXOSOMES	3	44	331	27.15	2017.87
METASTASIS	3	38	79	22.47	2017.95
PROTEOMICS	3	40	18	16.83	2018.28
TUMOR MICROENVIRONMENT	3	28	49	35.24	2018.02
BIOMARKERS	4	21	31	26.81	2017.48
CIRCULATING TUMOR CELLS	4	12	16	15.06	2018.19
DRUG DELIVERY	4	14	21	46.81	2018.19
EXTRACELLULAR VESICLES	4	39	164	14.53	2018.22
HER2	4	9	10	17.10	2018.30
LIQUID BIOPSY	4	17	47	10.83	2019.17
TRIPLE-NEGATIVE BREAST CANCER	4	12	11	23.27	2018.91
CANCER-ASSOCIATED FIBROBLASTS	5	9	10	25.60	2018.90
DRUG RESISTANCE	5	21	29	23.69	2017.76
MICROENVIRONMENT	5	15	11	55.82	2016.36
MICRORNAS	5	21	27	40.89	2017.48
TUMOR	5	13	12	36.00	2017.50
BIOMARKER	6	20	52	28.58	2018.12
EXOSOME	6	37	156	31.64	2017.77
EXTRACELLULAR VESICLE	6	19	31	19.77	2017.74
MICRORNA	6	28	64	34.56	2017.80
SERUM	6	12	12	31.92	2017.25
